# An experimental model of Braak’s pretangle proposal for the origin of Alzheimer’s disease: the role of locus coeruleus in early symptom development

**DOI:** 10.1186/s13195-019-0511-2

**Published:** 2019-07-03

**Authors:** Abhinaba Ghosh, Sarah E. Torraville, Bandhan Mukherjee, Susan G. Walling, Gerard M. Martin, Carolyn W. Harley, Qi Yuan

**Affiliations:** 1 Division of Biomedical Sciences, Faculty of Medicine, Memorial University of Newfoundland, St. John’s, NL A1B 3V6 Canada; 2 Department of Psychology, Faculty of Science, Memorial University of Newfoundland, St. John’s, NL A1B 3X9 Canada

**Keywords:** Locus coeruleus, Norepinephrine, Hyperphosphorylated tau, Pretangles, Odor discrimination, Odor identification deficit

## Abstract

**Background:**

The earliest brain pathology related to Alzheimer’s disease (AD) is hyperphosphorylated soluble tau in the noradrenergic locus coeruleus (LC) neurons. Braak characterizes five pretangle tau stages preceding AD tangles. Pretangles begin in young humans and persist in the LC while spreading from there to other neuromodulatory neurons and, later, to the cortex. While LC pretangles appear in all by age 40, they do not necessarily result in AD prior to death. However, with age and pretangle spread, more individuals progress to AD stages. LC neurons are lost late, at Braak stages III–IV, when memory deficits appear. It is not clear if LC hyperphosphorylated tau generates the pathology and cognitive changes associated with preclinical AD. We use a rat model expressing pseudohyperphosphorylated human tau in LC to investigate the hypothesis that LC pretangles generate preclinical Alzheimer pathology.

**Methods:**

We infused an adeno-associated viral vector carrying a human tau gene pseudophosphorylated at 14 sites common in LC pretangles into 2–3- or 14–16-month TH-Cre rats. We used odor discrimination to probe LC dysfunction, and we evaluated LC cell and fiber loss.

**Results:**

Abnormal human tau was expressed in LC and exhibited somatodendritic mislocalization. In rats infused at 2–3 months old, 4 months post-infusion abnormal LC tau had transferred to the serotonergic raphe neurons. After 7 months, difficult similar odor discrimination learning was impaired. Impairment was associated with reduced LC axonal density in the olfactory cortex and upregulated β1-adrenoceptors. LC infusions in 14–16-month-old rats resulted in more severe outcomes. By 5–6 months post-infusion, rats were impaired even in simple odor discrimination learning. LC neuron number was reduced. Human tau appeared in the microglia and cortical neurons.

**Conclusions:**

Our animal model suggests, for the first time, that Braak’s hypothesis that human AD originates with pretangle stages is plausible. LC pretangle progression here generates both preclinical AD pathological changes and cognitive decline. The odor discrimination deficits are similar to human odor identification deficits seen with aging and preclinical AD. When initiated in aged rats, pretangle stages progress rapidly and cause LC cell loss. These age-related outcomes are associated with a severe learning impairment consistent with memory decline in Braak stages III–IV.

**Electronic supplementary material:**

The online version of this article (10.1186/s13195-019-0511-2) contains supplementary material, which is available to authorized users.

## Background

In a survey of 2332 human brains aged from 1 to 100, Braak and colleagues reported that the first evidence of soluble abnormal or hyperphosphorylated tau, which appears later in association with the cortical tangles of Alzheimer’s disease (AD), is in the locus coeruleus (LC) neurons of the brain stem [1]. Importantly, this abnormal tau appears even at young ages. Five pretangle tau stages were identified in the human brain [2]: (a) abnormal tau in LC axons, (b) in LC axons and the somatodendritic LC compartment, and (c) in the foregoing and other neuromodulatory cell groups, such as the serotonergic raphe nuclei; (1a) along LC axons to their terminals in transentorhinal/entorhinal cortex; (1b) in the pyramidal cells of transentorhinal cortex. When Braak’s AD diagnostic *insoluble* tau tangle stages (Braak I–VI) appear, the pretangle stages are still present [3]. Pretangle stages a–c only, predominate at ages 10–20, 1a–1b appear mainly at ages 40–50, while from age 60 onwards, Braak tangle stages I–II are more frequently observed, followed by symptomatic AD stages III–VI in the 80–100 age range. In these later stages, LC neurons themselves are lost [4]. While Braak’s model appears compelling, some investigators [5] have assumed that, as pretangle stages are ubiquitous in the human brain, they are unlikely to be the driving source of AD and note, further, that there is no evidence that pretangles can generate AD phenomenology. Here, we use an animal model to ask if LC pretangles, in the absence of any amyloid, can generate functional and anatomical pathology characteristic of preclinical AD descriptions. This outcome would support the hypothesis that LC pretangles are AD ground zero.

The work of Braak and others [6, 7] demonstrates soluble tau pretangle expression in the LC neurons and, subsequently, in other subcortical nuclei [8] and in the entorhinal cortex. The earliest tangles reported are associated with the anterior olfactory nucleus and entorhinal cortex [9, 10]. The anterior olfactory nucleus is a component of the human olfactory cortex (prepiriform/piriform) [11]. Both the entorhinal cortex and the anterior olfactory nucleus receive direct projections from the olfactory bulb [12].

Our work on LC function in rats, and its critical role in highly challenging olfactory discrimination tasks [13], led us to hypothesize that pretangle stages characterized, initially, by persistent hyperphosphorylated tau expression in the LC neurons may explain the early appearance of human odor identification deficits with aging [14] since Braak’s data argues that all aging humans will have some hyperphosphorylated abnormal tau in the LC neurons. Worsening olfactory deficits are subsequently predictive of MCI development, when LC neurons are likely to begin to be lost, while yet greater olfactory identification impairment deficits predict AD itself [15–28]. Importantly, olfactory identification deficits, as we hypothesize, have been shown to appear in a cognitively normal population prior to the appearance of episodic memory deficits. In a longitudinally studied population, the early identification deficits predicted the subsequent rate of episodic memory decline [29]. Decreases in human olfactory identification ability have been related to tau pathology [30], to impairments in odor coding [31], and to hypometabolism in primary olfactory structures including the piriform cortex [32, 33]. The identification of the precise mechanistic underpinning of early olfactory dysfunction may be critical for early intervention to prevent the development of AD.

In the present study, we generate an animal model to test the hypothesis that the expression of persistently phosphorylated tau in the LC neurons will lead initially to impairments in associative olfactory learning and memory. Our model is generated by infusing a human pseudophosphorylated tau (htau) gene into the LC. Pseudophosphorylation functionally mimics soluble persistently phosphorylated tau [34]. Although Braak used an antibody (AT8) to two tau phosphorylation sites to identify pretangle tau, a recent examination of human LC neurons displaying abnormal tau [35] used additional antibodies and reported another 5 persistently phosphorylated sites. These seven sites are all proline-directed serine/threonine sites. Normal tau has 87 phosphorylation sites available, but those identified with early AD are typically the proline-directed sites. Goedert proposed that “while normal brain tau is phosphorylated at only a few of the 17 serine/threonine-proline sites, (AD) tau is phosphorylated at a large number of these sites” [36]. The phosphorylation of AD-related soluble and insoluble tau survives death and fixation unlike the phosphorylation of normal tau; for this reason, we use the phrase “persistently phosphorylated.” Persistent phosphorylation argues that the normal dephosphorylation mechanism is not effective for pretangle tau. Karen Ashe has provided a plasmid for human tau on Addgene with 14/17 proline-directed sites pseudophosphorylated (htauE14) that produces, functionally, persistent phosphorylation of those sites. Since persistent phosphorylation of proline-directed sites in LC pretangles is characteristic, this led us to choose the Ashe plasmid for E14 tau for insertion in the LC neurons. Human LC pretangle neurons also show an increase in 4R isoforms [37], which is the backbone of the Addgene E-14 tau (0N/4R).

Here, we inserted htauE14 in the LC to initiate the human soluble hyperphosphorylated pretangle stages in tyrosine hydroxylase (TH)-Cre rats. We subsequently tested these rats for their ability to perform simple and difficult odor discriminations. We also examined the spread of human tau along the LC axons, LC neuron density, and fiber density in the primary olfactory (piriform) cortex. We correlated behavioral deficiency with LC pathophysiology at different stages in rats.

## Methods

### Ethics statement and subjects

Experiments were conducted following the guidelines of the Canadian Council of Animal Care. Experimental protocols were approved by the Memorial University Institutional Animal Care Committee. TH-Cre male rats (Sage) were bred with Sprague-Dawley (SD) female rats to generate the heterozygous offspring used in the experiments. Forty-four TH-Cre and twelve SD rats of both sexes were used in this study. Rats were housed on a 12-h light/dark cycle with ad libitum access to water and dry food. Water deprivation started 5 days before the behavioral tests in an olfactometer. During that period, rats had access to either ad libitum water for an hour per day or a total volume of 25 ml water per day.

### Experimental design and statistical analysis

**Fig. 1:**
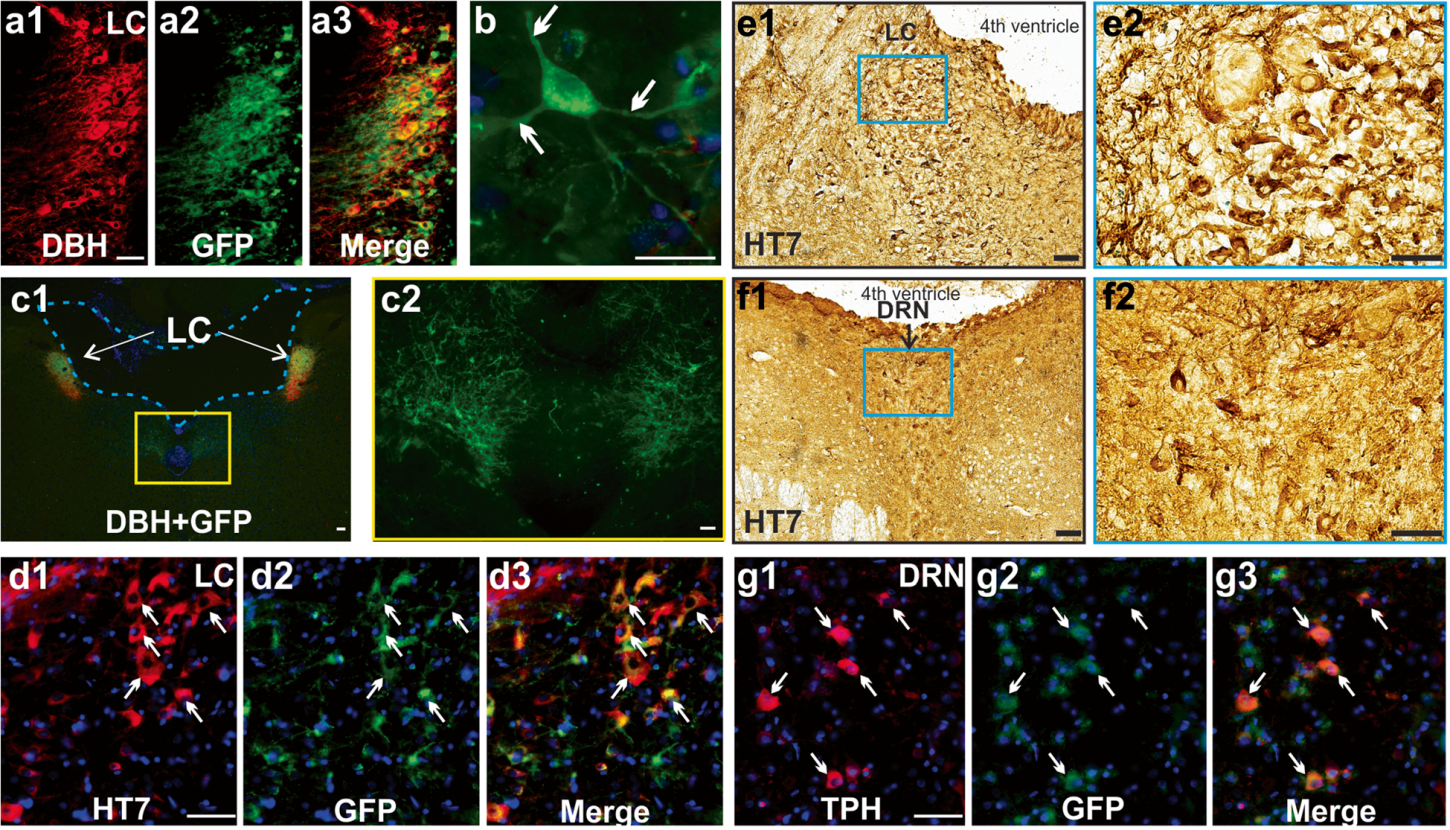
HtauE14 uptake in the LC and spread. **a1**–**a3** Expression of htauE14 (green) in DBH (red) LC neurons in a rat. **b** Somatodendritic mislocalization of htauE14 in an LC neuron (arrows). **c1** Spread of htauE14 to the midline brain stem in an 8-month-old rat (12 weeks post-infusion). **c2** Enlargement of GFP cells in **c1** (yellow box). **d1**–**d3** HT7 (red) expression in the LC in a 19-month-old rat (7 months post-infusion). Arrows indicate GFP and HT7 co-expressed cells. **e1**–**e2** HT7 staining in the LC in the same rat as in **d**. **f1**–**f2** Midline HT7^+^ dorsal raphe neurons (DRN). **g1**–**g3** Co-expression of GFP and TPH in cells (indicated by arrows) in the midline. Scale bar, 50 μm

**Fig. 2:**
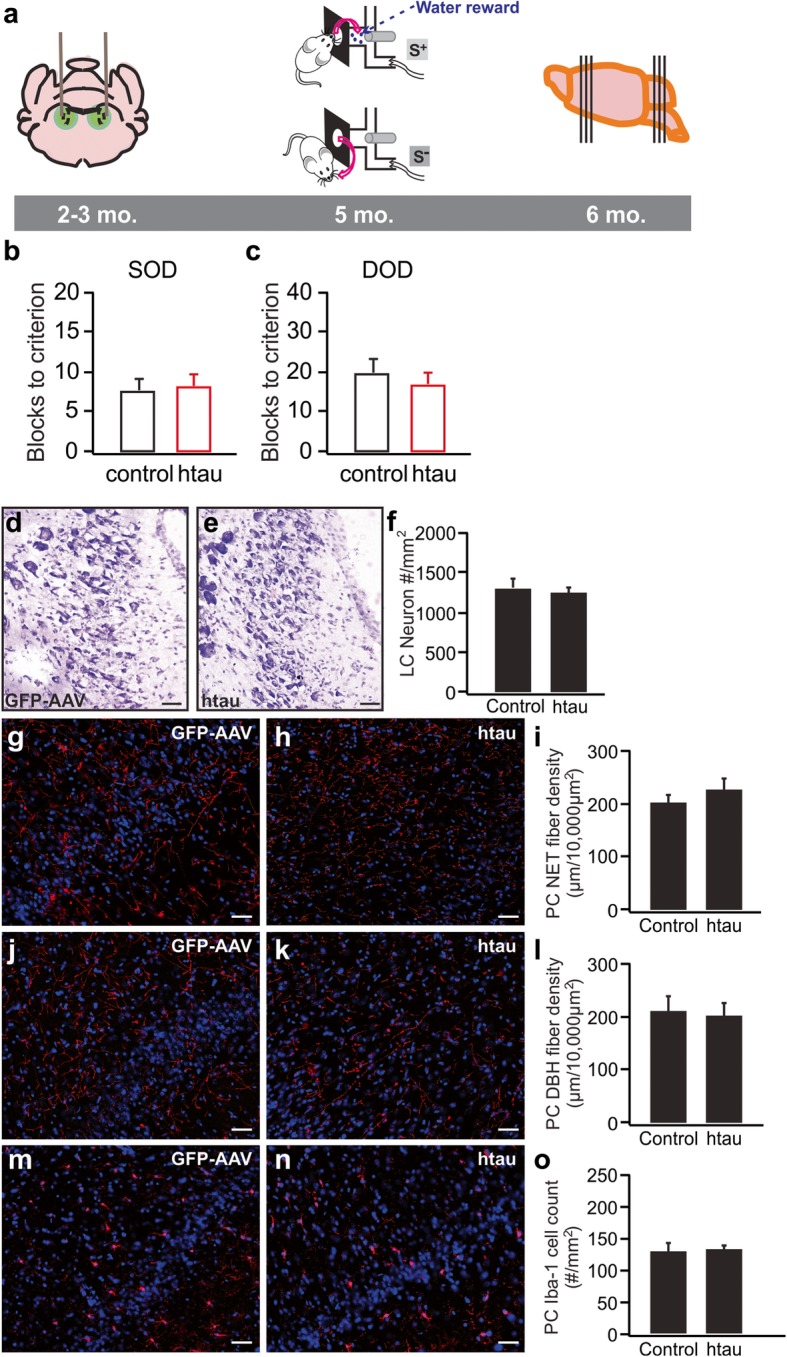
No odor discrimination deficiency or LC degeneration in young 6-month-old rats infused with htauE14 at 2–3 months old. **a** Schematics of the time course of the AAV infusion, odor discrimination training, and histology. **b** Simple odor discrimination (SOD) training using dissimilar odors. **c** Difficult odor discrimination (DOD) training using similar odor pairs. (N: htau/control: 6/4). **d**–**f** LC cell counts in htauE14 and control rats. (N: htau/control: 5/5). **g**–**i** NET fibers (red) in the piriform cortex (PC). (N: htau/control: 6/5). **j**–**l** DBH fibers (red) in the PC. (N: htau/control: 6/5). **m**–**o** Iba-1 cell staining (red) in the PC. (N: htau/control: 6/5). Scale bars, 50 μm

**Fig. 3:**
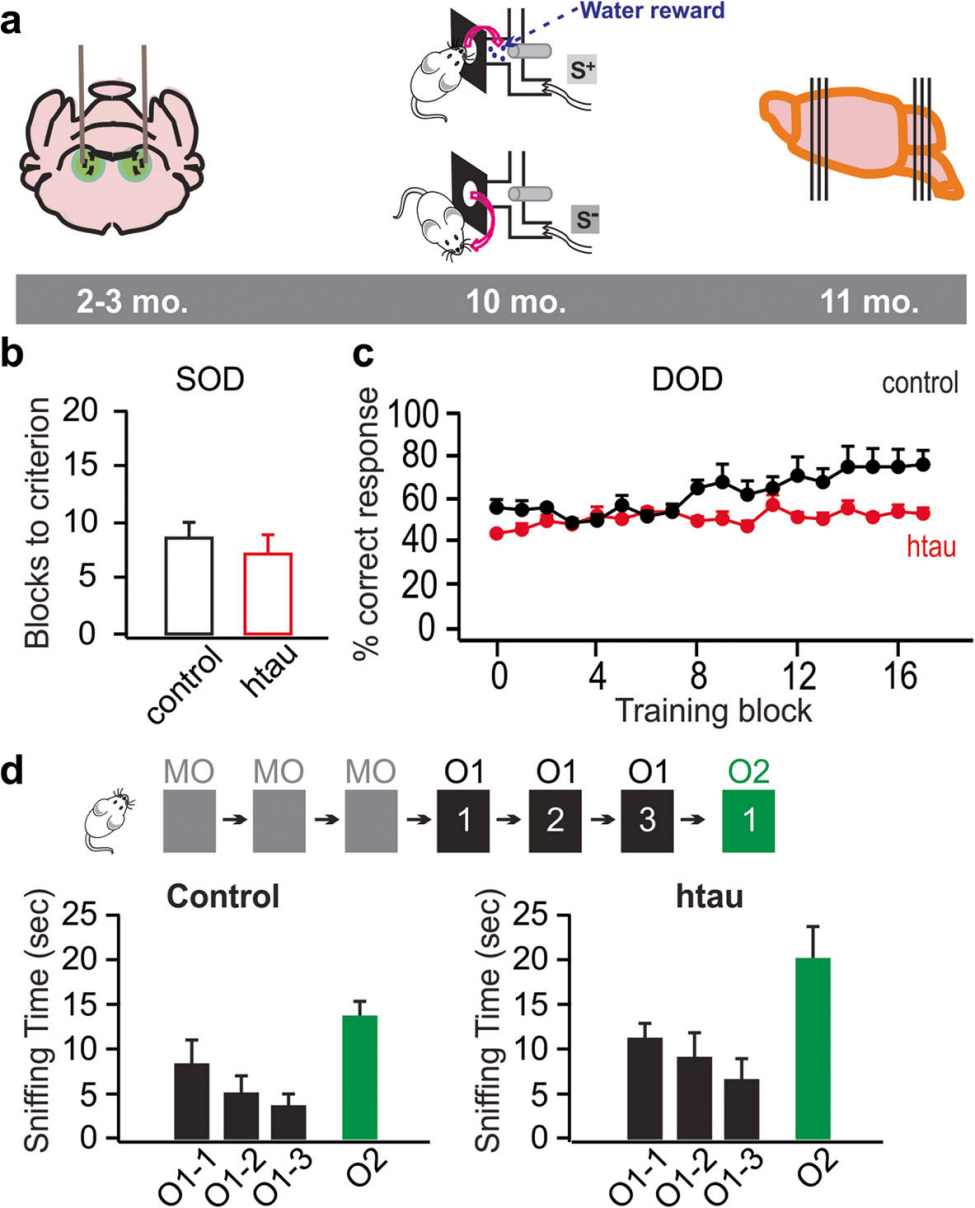
Impairment in go/no-go odor discrimination learning in 10-month-old rats infused with htauE14 at 2–3 months old. **a** Schematics of the time course of the AAV infusion, odor discrimination training, and histology. **b** Simple odor discrimination (SOD) training using dissimilar odors. (N: htau/control: 7/7). **c** Difficult odor discrimination (DOD) training using similar odor pairs. (N: htau/control: 6/5). **d** Odor habituation/dishabituation test. (N: htau/control: 6/7). MO, mineral oil. O1, odor 1. O2, odor 2

**Fig. 4:**
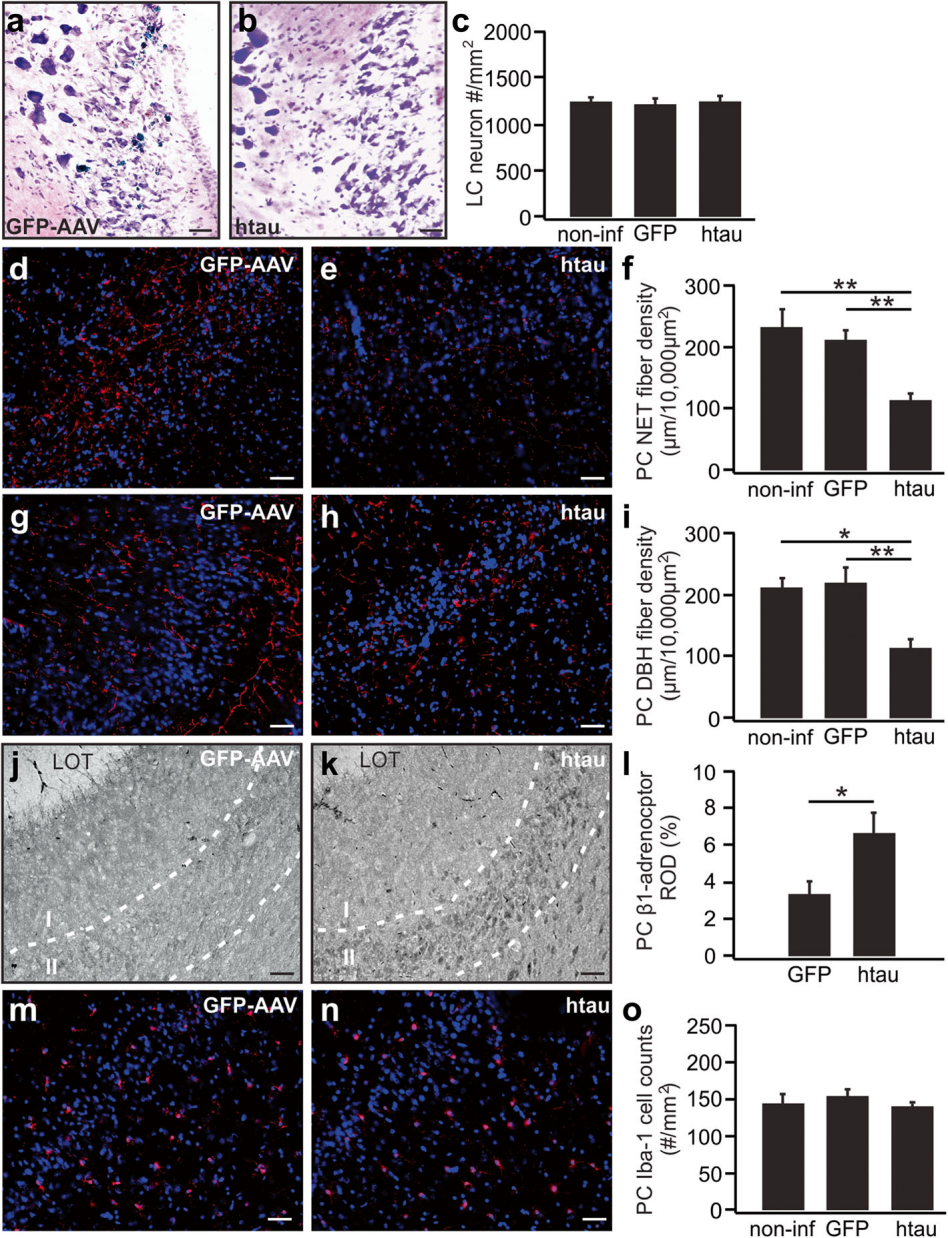
LC axonal degeneration in 11-month-old rats infused with htauE14 at 2–3 months old. **a**–**c** LC cell counts in htauE14 and control rats. (N: htau/GFP/non-infused: 7/6/6). **d**–**f** NET fibers (red) in the piriform cortex (PC) (N: htau/GFP/non-infused: 6/6/5). **g**–**i** DBH fibers (red) in the PC. (N: htau/GFP/non-infused: 6/5/5). **j**–**l** β1-adrenoceptors in the PC. (N: htau/GFP: 4/4). **m**–**o** Iba-1 in the PC. (N: htau/GFP/non-infused: 6/5/6). LOT, lateral olfactory tract. Scale bars, 50 μm

**Fig. 5:**
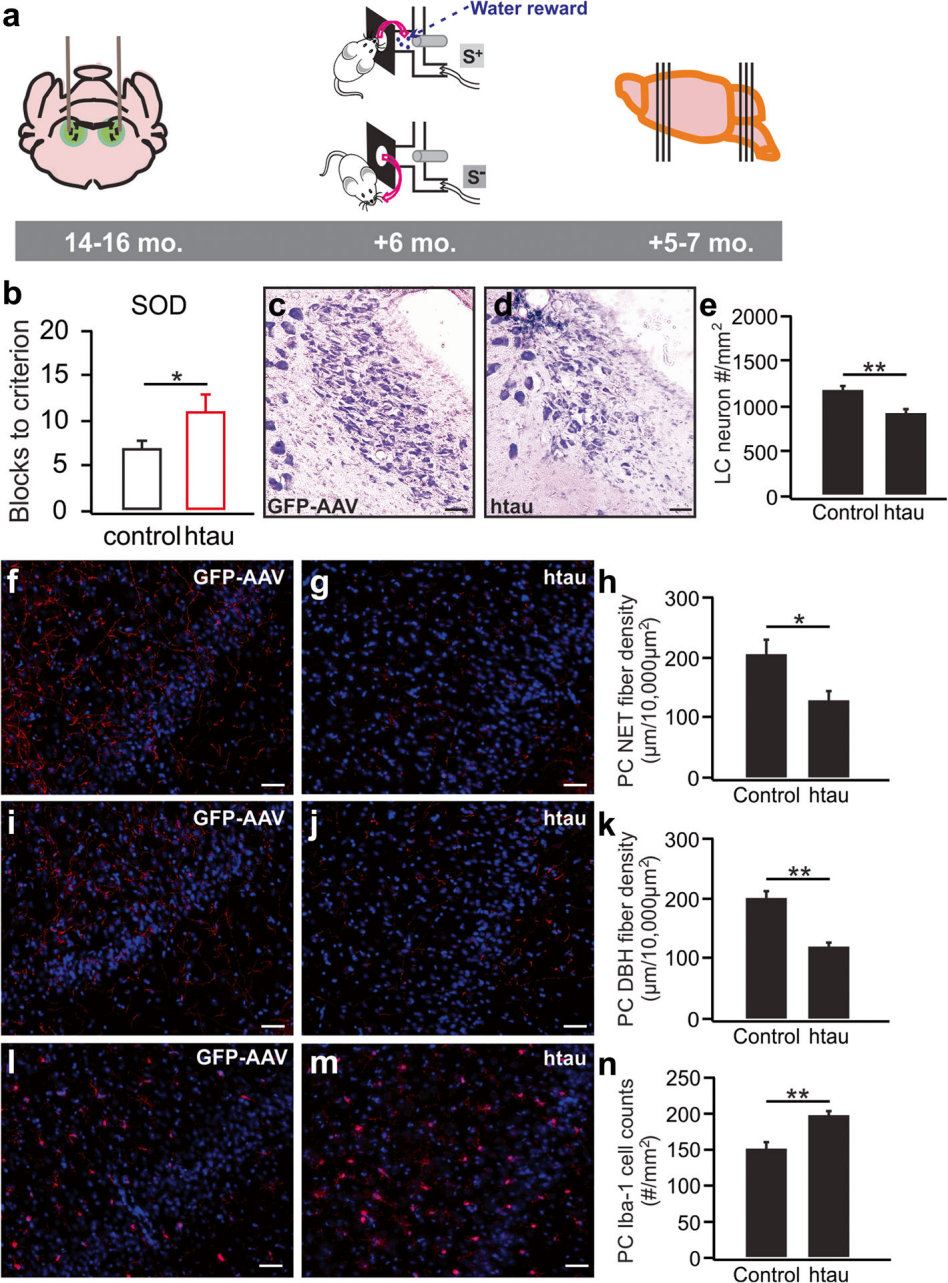
Deficiency in odor discrimination and LC degeneration in 17–20-month-old rats infused with htauE14 at 12–14 months old. **a** Schematics of the time course of the AAV infusion, odor discrimination training, and histology. **b** Simple odor discrimination (SOD) training using dissimilar odors. (N: htau/GFP: 4/7). **c**–**e** LC cell counts in htauE14 and control rats. (N: htau/GFP: 6/5). **f**–**h** NET fibers (red) in the piriform cortex (PC). (N: htau/GFP: 6/6). **i**–**k** DBH fibers (red) in the PC. (N: htau/GFP: 6/6). **l**–**n** Iba-1 staining in the PC. (N: htau/GFP: 6/6). Scale bars, 50 μm

**Fig. 6:**
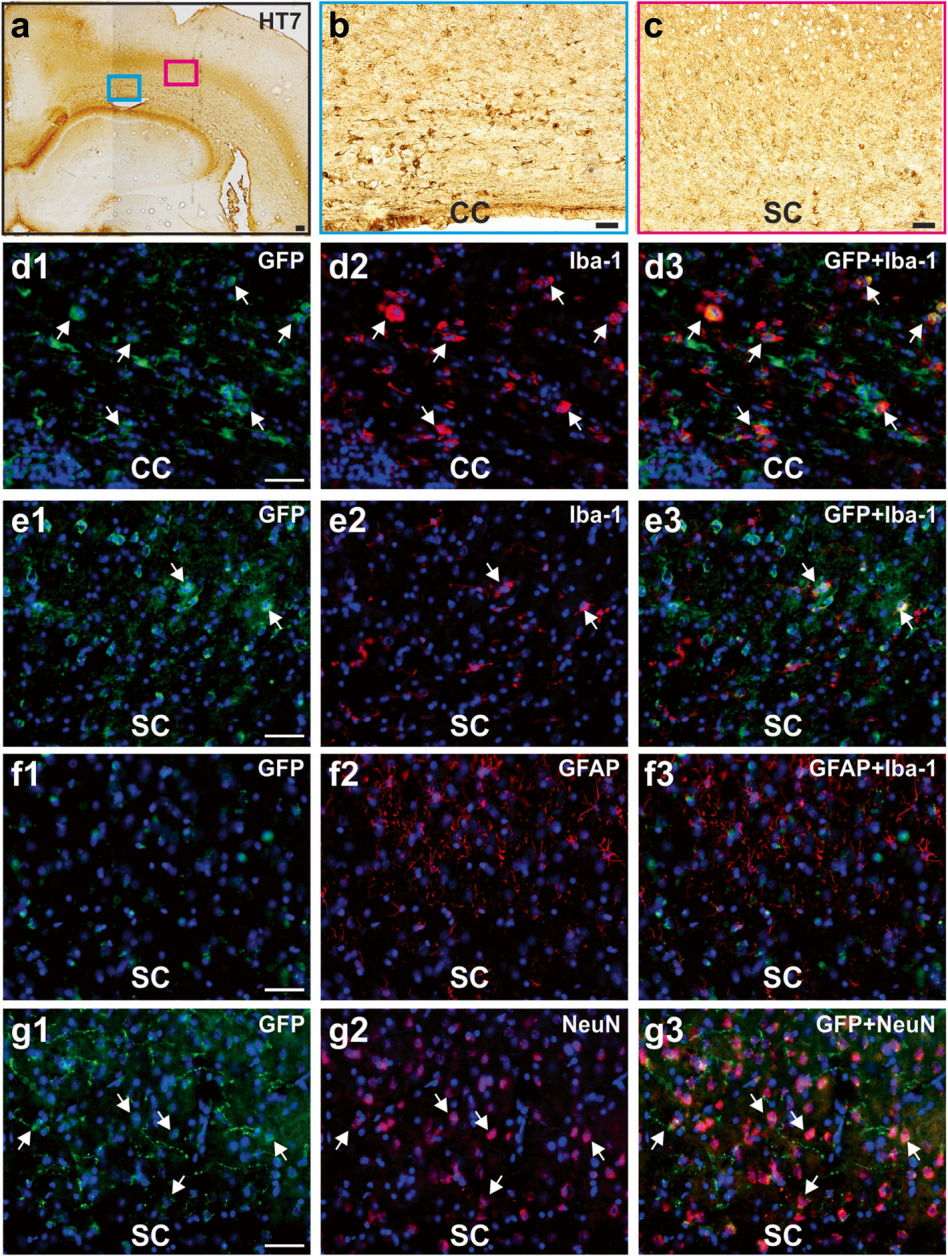
HtauE14 release and uptake by neurons and microglia in an old AD rat. **a** HT7 staining in a 21-month-old rat, 7 months post-infusion. Scale bar, 200 μm. **b** Enlargement of a region in the corpus callosum (CC) in A. **c** Enlargement of a region in the sensory cortex (SC) in A. **d1**–**d3** GFP^+^ cells in the CC and some co-localization with Iba-1^+^ microglia. **e1**–**e3** GFP^+^ cells in the sensory cortex (SC) and some co-localization with Iba-1^+^ microglia. **f1**–**f3** No co-localization of GFP and GFAP^+^ cells. **g1**–**g3** GFP^+^ cells in the SC co-localized with NeuN. Scale bars, 50 μm

We first characterized AAV uptake and htauE14 expressions in the LC and spread to the serotonergic raphe neurons in our TH-Cre rats (Fig. 1). We then conducted three experiments. In the first experiment, rats were infused with htauE14 AAV or control GFP-only AAV at 2–3 months of age, trained in the odor discrimination learning task at 2–3 month post-infusion (~ 5 month of age), and sacrificed for brain histology and immunohistochemistry following the behavioral task (~ 6 month of age) (Fig. 2). In the second experiment, rats were infused at 2–3 months of age and trained in the olfactory tasks 7–8 months later (~ 10 months of age), followed by brain processing (~ 11 months of age) (Figs. 3 and 4). A subset of rats underwent either behavioral training or immunohistochemistry, but not both at these time points. In the third experiment, rats were infused at 14–16 months of age and trained in the olfactory discrimination task 6 months later (~ 20–22 months of age). Brain histology and immunohistochemistry were conducted 5–7 months post-infusion (Figs. 5 and 6). A cohort of non-infused rats was used as a control in different age groups as well.

Two-way mixed ANOVAs followed by linear trend analyses were used to determine statistical significance for the difficult odor discrimination experiment and the habituation/dishabituation experiment. One-way ANOVAs and post-hoc Tukey’s tests were applied to the experiments with > 2 group comparisons. All two group comparisons were subjected to Student’s *t* tests (unpaired, two-tail). Data are presented as mean ± SEM.

The primary outcome measure was the number of trials to criterion for the difficult similar odor discrimination task. Based on our previous investigation of such learning with adrenergic receptor blockade in the piriform cortex in rats [13], an *n* of 4 rats/group is sufficient to detect normal acquisition of a difficult odor discrimination with a power of 0.74. Accordingly, all odor learning experiments had group sizes of at least 4/group.

### Viral transduction of LC neurons by stereotaxic surgery

We used a human tau construct in which 14 proline-directed phosphorylation sites were substituted with glutamate to produce a pseudophosphorylated tau (htauE14) [1]. Adeno-associated virus 9 (AAV9) was used as a vector (AAV9-rEF1a-DIO-EGFP-htauE14; 2.26E^+ 13^ vg/ml, MIT). The EGFP-htauE14 expression cassette was placed under a double inverted open reading frame (DIO) for Cre-dependent expression. Control virus lacked htauE14 (AAV9-rEF1a-DIO-EGFP, 2.35E^+ 13^ vg/ml). Under isofluorane anesthesia, TH-Cre rats were placed in a stereotaxic. One microliter of virus mixed with 0.4 μl fluorescent beads (0.1%) was infused in each LC through an infusion pump and a guide cannula which was placed in a parasagittal plane at an angle of 20° caudal to the coronal plane. LC coordinates were 11.8–12.2 mm posterior, 1.3 mm bilateral, and 6.3 mm ventral with respect to the bregma.

### Behavioral testing

#### Odor discrimination learning

Rats underwent go-no go odor discrimination training in a four-channel Knosys olfactometer as described previously [13, 38]. Initially, rats were trained to lick a water delivery port for a reward of a 30-μl drop/lick following 1% orange odor presentation (S^+^). Duration of the trial was 2.5 s with the first 0.5 s for odor presentation. A minimum of 6 licks delivered the reward. The inter-trial interval was 5 s. Each rat underwent approximately 100 trials per day for 3 days before moving on to the simple odor discrimination phase, in which a 2% peppermint odor (S^−^), not associated with reward, was added. In blocks of 20 trials, S^+^ and S^−^ odors were presented an equal number of times in a random order. Each rat went through 5–10 blocks of training each day, until they reached a learning criterion of 3 consecutive blocks with at least 70% correct. Following successful simple odor discrimination, rats underwent difficult odor discrimination training in which a pair of similar odors had to be discriminated in order to get water reward. 0.001% heptanol vs. 0.001% heptanol and octanol at a 1:1 ratio were used as the difficult odor pair. Rats were trained until the learning criterion was reached or until a certain number of blocks were completed. Odorants were prepared freshly for each experiment in 10 ml of mineral oil solvent.

#### Acute odor habituation/dishabituation test

In a modified version of a detection test described by Escanilla et al. [39], rats were tested with similar odors presented on sponges (O1 = 0.001% heptanol; O2 = 0.001% heptanol and octanol at 1:1 ratio). Following a 5-min habituation period inside a semi-transparent plastic box (600 x 600 x 600 mm^3^), rats were presented with an odor-free sponge at one corner of the box for another 5 min. After that, 7 trials of sponge presentation took place, of which the first 3 contained mineral oil, second 3 contained O1, and the last one contained O2. Each trial lasted 50 s with 5 min inter-trial intervals. Each sponge contained 60 μl of odorant or mineral oil. Rat’s sniffing time within a radius of 1 cm around the sponge was videotaped and measured offline during O1 and O2.

### Histology and immunohistochemistry

Rats were anesthetized by intraperitoneal injection of a 50-mg/kg pentobarbital solution followed by transcardiac perfusion of 0.9% saline and 4% paraformaldehyde (PFA) respectively. The brains were extracted and left in PFA overnight at 4 °C and then transferred to a 20% sucrose solution the next day. Thirty-micrometer slices were cut in a Leica cryostat and collected on gelatin-coated glass slides for further processing.

For Nissl staining, the slides were brought to room temperature, and rehydration was performed by dipping the slides in decreasing concentrations of ethanol (100%, 95%, and 70%) for 3 min each and deionized water for 1 min followed by an aqueous solution of 0.5% cresyl violate acetate (Sigma) for 8 min. After 1 min in deionized water, the slides were dehydrated in increasing concentrations of ethanol (70%, 95%, and 100%) for 3 min each. Afterwards, a quick clearing step was performed by dipping the slides in xylene for 1 min. The slides were then coverslipped using Permount (Fisher Scientific).

Immunohistochemistry was performed by incubating the slides inside a humidified chamber overnight at 4 °C in primary antibodies (see Table 1), which were dissolved in phosphate-buffered saline (PBS) and mixed with 2% normal goat serum and 0.25% Triton X-100. For fluorescence staining, after a 30-min wash in PBS, the slides were incubated in suitable secondary antibody (see Table 1) for 1 h at room temperature followed by a 30-min wash in PBS and mounting with DAPI. For amplification, following overnight primary antibody incubation, the slides were washed in PBS for 30 min and incubated in a suitable biotinylated secondary antibody (see Table 1) for 1 h. After a 30-min wash in PBS, an avidin and biotinylated enzyme amplification step (A+B) was applied. In the end, color was developed by a diaminobenzidine tetrahydrochloride (DAB) reaction [50 mg DAB (Amresco) + 50 ml PBS + 50 ml water + 30 μl 30% H_2_O_2_]. Antigen retrieval was performed by heating the slides in 10 mM sodium citrate buffer containing 0.05% Tween (pH adjusted to 6.0) at 90 °C for 15 min before incubating with tryptophan hydroxylase (TPH), NeuN, and β1-adrenoceptor primary antibodies.

**Table 1:** List of antibodies

Primary antibody	Company/product number	Primary antibody		Secondary antibody (fluorescence)		Secondary antibody (biotinylation amplification)	
		Host	Conc.	Company/product	Conc.	Company/product	Conc.
Iba-1	Wako/019-19741	Rabbit	1:1000	Invitrogen/A31572	1:500	–	–
β1 AR	Abcam/ab3442	Rabbit	1:400	–	–	Vector/BA 1000	1:500
NET	Invitrogen/MA5-24647	Mouse	1:500	Invitrogen/A31570	1:500	–	–
DBH	Millipore/MAB308	Mouse	1:500	Invitrogen/A31570	1:500	–	–
TPH	Sigma/T0678	Mouse	1:400	Invitrogen/A31570	1:500	Vector/BA 9200	1:500
NeuN	Millipore/MAB 377	Mouse	1:100	Invitrogen/A31570	1:500	–	–
HT7	Invitrogen/MN1000	Mouse	1:500	Invitrogen/A31570	1:500	Vector/BA 9200	1:1000
GFP	Thermo Fisher/A11122	Rabbit	1:200	Invitrogen/A27034	1:500	–	–
AT8	Thermo Fisher/MN1020	Mouse	1:200	–	–	Vector/BA 9200	1:1000

*Iba-1* ionized calcium-binding adaptor molecule 1, *AR* adrenoceptor, *NET* norepinephrine transporter, *DBH* dopamine beta-hydroxylase, *TPH* tryptophan hydroxylase, *NeuN* neuronal nuclei, *GFP* green fluorescence protein, *HT7* human tau 7, *AT8* anti-tau 8

#### Thioflavin-S staining

Slides were quenched of GFP signal first by heating at 90 °C for 10 min. After 5 min of defatting in xylene, slides were rehydrated in serial dilutions of ethanol (100%, 95%, 70%, 50%; 3 min each) and water (2 × 3 min). Then slides were incubated for 8 min in a filtered 1% aqueous solution of Thioflavin-S (Sigma) in the dark at room temperature. Washes with ethanol (70%, 2x3min; 95%, 3 min) and water (3 exchanges) followed before mounting with aqueous mounting media (Sigma).

### Imaging analysis and quantification

Bright-field and fluorescence images were acquired by Apotome 2 (Zeiss) and BX-51 (Olympus). Images were analyzed with ImageJ software. For LC fiber density, images were background subtracted and converted into binary images. Fiber length was calculated using the DiameterJ plugin. For LC cell counts and Iba-1^+^ cell count in the piriform cortex, the population density of neurons (number of neurons per unit area) was calculated and averaged over 3–6 sections rostral to caudal in each rat. LC neurons were identified from the Nissl stains following Garcia-Cabezas et al. [40]. For β1-adrenoceptor density in the piriform cortex, a region of interest (ROI) was manually traced over the layer II pyramidal cells of the anterior piriform cortex ventral to the lateral olfactory tract. An optical density (OD) reading of the background was taken in layer Ia for comparison. The relative OD (ROD) was calculated using the following formula: ROD = |(OD of ROI − OD of background)/OD of background|. RODs from 3 to 4 sections were averaged for each animal.

## Results

### HtauE14 uptake in the LC and spread

LC neurons co-expressed htauE14 within 1 week of infusion (Fig. 1a1–a3) and htauE14 became mislocalized to the LC cell bodies and dendrites (Fig. 1b) similar to the pathology in pretangle AD [1] and the htauE14 mislocalization reported in hippocampal neurons [34]. The transfection rate indexed by GFP^+^ cells over DBH^+^ LC neurons is 83.0 ± 4.3% in htauE14 rats (*n* = 9). The targeting to LC is shown in Additional file 1. At 6 weeks, htauE14 spread along LC axons toward forebrain targets, and by 12 weeks, htauE14 reached its furthest target, the olfactory bulb and other cortical areas such as the hippocampus and the piriform cortex (Additional file 2). At the same time, the spread of the GFP signal to pontine midline neurons was observed (Fig. 1c1, c2). Using an antibody HT7 which detects human tau, we confirmed human tau co-localized with GFP in the LC (Fig. 1d1–d3) and HT7 could be seen with light microscopy in the LC (Fig. 1e1, e2). HT7 was also observed to have spread to putative raphe midline neurons (Fig. 1f1, f2) as seen in humans [8, 41]. The location of midline raphe neurons in rats is indicated by TPH staining (Additional file 3). We confirmed GFP co-localization in raphe neurons with TPH (Fig. 1g1–g3).

We have attempted to index tau hyperphosphorylation with the AT8 antibody. However, staining was not observed (Additional file 4). Some phosphorylation site-specific antibodies such as AT8 typically do not recognize sites that are pseudophosphorylated [42]. Nevertheless, we could demonstrate that pseudophosphorylated human tau was expressed in our tissue by the HT7 antibody.

### HtauE14 is expressed in young adult LC neurons and spreads transneuronally to the raphe by 3–4 months post-infusion but does not produce olfactory discrimination impairments

In humans, AD-associated soluble persistently phosphorylated tau as indexed by the AT8 antibody first appears in the LC in a minority of individuals prepubertally [2] but is observed nearly universally by 40 years of age [1, 3]. Despite the prevalence of pretangle tau in LC, the early effects of this tauopathy in middle-aged humans are unknown. Our model mimics the early expression of soluble persistently phosphorylated tau in LC through the infusion of htauE14 in 2–3-month-old rats. We subsequently first examined physiological and cognitive changes in adult rats 5–6 months old (3–4 months post-infusion; Fig. 2), a time point at which, besides abnormal tau expression in LC and its projection along LC axons to the olfactory cortex (piriform), transfer of abnormal tau to other midline brain stem cell groups have been observed. Both htauE14 rats and control rats were subjected to odor discrimination training 3 months post-infusion, and brain histology and immunohistochemistry were conducted at 4 months post-infusion, following the odor discrimination task (Fig. 2a).

The htauE14 rats showed no deficiency in either dissimilar (no. of blocks to reach learning criterion 7.75 ± 1.25, *n* = 4 in control vs. 8.33 ± 1.28, *n* = 6 in htauE14, *t* = 0.31, *p* = 0.76; Fig. 2b), or similar odor discrimination learning (no. of blocks to reach learning criterion 20.50 ± 2.60, *n* = 4 in control vs. 17.17 ± 2.48, *n* = 6 in htauE14, *t* = 0.89, *p* = 0.40; Fig. 2c). In parallel, htauE14 rats showed no LC cell loss (htauE14 1276.2 ± 38.6/mm^2^, *n* = 5 vs. control 1339.4 ± 86.8/mm^2^, *n* = 5, *t* = 0.67, *p* = 0.52; Fig. 2d–f), no difference in norepinephrine transporter (NET) fiber density in the piriform cortex (htauE14 227 ± 21.3 μm/10,000 μm^2^, *n* = 6 vs. control 204.6 ± 12.5 μm/10,000 μm^2^, *n* = 5, *t* = 0.85, *p* = 0.41; Fig. 2g–i), and no difference in DBH fiber density (htauE14 203.7 ± 21.9 μm/10,000 μm^2^, *n* = 6 vs. control 212.8 ± 26.2 μm/10,000 μm^2^, *n* = 5, *t* = 0.27, *p* = 0.79; Fig. 2j–l). Iba-1 staining was not different in the two groups (htauE14 132.2 ± 6.7 #/mm^2^, *n* = 6 vs. control 131.2 ± 12.8 #/mm^2^, *n* = 5, *t* = 0.07, *p* = 0.94; Fig. 2m–o). Together, these results suggested that the initial pretangle stages are not linked to the difficulties in odor discrimination, consistent with the lack of olfactory identification deficits in humans in a preclinical stage, despite pretangle abnormal tau being present universally at age 40.

### HtauE14 expression in young adult LC neurons results in LC fiber degeneration in the piriform cortex and impairment in difficult odor discrimination learning by 7–8 months post-infusion

We next examined rats that were 7–8 months post-infusion (Fig. 3a). Unlike the earlier time point, by 7 months post-infusion, htauE14 rats did exhibit deficiency in difficult, similar odor discrimination learning. While there was no difference in simple, dissimilar odor discrimination learning (no. of blocks to reach learning criterion 8.6 ± 1.25, *n* = 7 in control vs. 7.1 ± 1.52, *n* = 7 in htauE14, *t* = 0.72, *p* = 0.48; Fig. 3b), we observed a significant impairment in difficult similar odor discrimination learning in the htauE14 rats compared to the same aged GFP control rats (Fig. 3c). A 2 (group) × 18 (block) ANOVA revealed that the htauE14 group performed more poorly than the control group in the difficult similar odor discrimination (*F*_1, 9_ = 30.319, *p* < 0.001). There was also a block effect (*F*_17, 153_ = 6.309) and a group × block interaction (*F*_17, 153_ = 4.237, *p* < 0.001). An analysis of the interaction revealed that the htauE14 group’s performance did not change over the 18 blocks (*F*_17,102_ = 1.145, *p* = 0.324) while the control group did differ over blocks (*F*_17, 51_ = 4.485, *p* < 0.001). Subsequent linear trend analysis showed that the control group improved over blocks (*F*_1, 3_ = 14.467, *p* = 0.032). This absence of a change in the htauE14 rats and improvement in the control rats shows that htauE14 LC expression impaired the capacity of the rats to learn a difficult odor discrimination relative to controls. Another cohort of rats, comparable in age and post-infusion times, was tested with the difficult odors used for the previous discrimination task in an odor habituation/dishabituation task (*n* = 7 control and *n* = 6 htauE14; Fig. 3d). A 2 × 3 ANOVA (group × odor presentation) revealed a significant effect of odor presentation (*F*_(2, 22)_ = 9.45, *p* < 0.001) but no group × odor presentation interaction (*F*_(2, 22)_ = 0.274, *p* = 0.763) or group effect (*F*_(1, 11)_ = 1.605, *p* = 0.231). The odor presentation effect reflected a habituation to the odor by both groups as indicated by a significant linear trend (*F*_(1, 11)_ = 17.639, *p* < 0.001). A 2 × 2 (group × last trial of odor 1 versus new odor 2) ANOVA revealed a significant difference between the odor 1 and new odor 2 (*F*_(1, 11)_ = 22.418, *p* < 0.001) and no group × odor interaction (*F*_(1, 11)_ = 0.570, *p* = 0.466) or group effect (*F*_(1, 11)_ = 4.114, *p* = 0.07). This pattern of results argues that the rats that failed the difficult discrimination learning were likely to have been able to detect the odor differences.

Nissl staining and cell counting of 11-month-old htauE14 and control rats (EGFP and non-infused) demonstrated no LC cell loss (*F*_(2, 16)_ = 0.17, *p* = 0.84; Fig. 4a–c), but significant NET fiber loss in the piriform cortex of the htauE14 rats indexed by either NET fiber density (*F*_(2, 14)_ = 12.43, *p* = 7.9E−4; Fig. 4d–f) or DBH fiber density in the piriform cortex (*F*_(2, 13)_ = 14.98, *p* = 4.2E−4; Fig. 4g–i). HtauE14 rats showed comparable levels of LC cell numbers (1277.7 ± 45.7/mm^2^, *n* = 7) to either GFP control rats (1235.8 ± 55.8/mm^2^, *n* = 6) or non-infused rats (1260.3 ± 52.3/mm^2^, *n* = 6). On the other hand, the htauE14 group showed a significant reduction in the NET fibers (111 ± 11.5 μm/10,000 μm^2^, *n* = 6) compared to either GFP controls (212.2 ± 16.4 μm/10,000 μm^2^, *n* = 6, *p* = 7.5E−4) or non-infused controls (233.2 ± 27.8 μm/10,000 μm^2^, *n* = 5, *p* = 0.001). Similarly, DBH fiber density was significantly lower in the htauE14 rats (114.8 ± 10.8 μm/10,000 μm^2^, *n* = 6) compared to either GFP controls (220.8 ± 21.9 μm/10,000 μm^2^, *n* = 5, p = 0.001) or non-infused controls (213.2 ± 14.1 μm/10,000 μm^2^, *n* = 5, *p* = 0.002). The LC axonal degeneration in the piriform cortex is accompanied by an upregulation of β1-adrenoceptors (ROD 0.07 ± 0.01 (htauE14) vs. 0.03 ± 0.005 (GFP), *n* = 4, *t* = 2.52, *p* = 0.045; Fig. 4j–l). However, Iba-1 staining of the microglia in the piriform cortex indicated no elevated microglia in the piriform cortex (*n* = 6 htauE14; *n* = 5 GFP; *n* = 6 non-infused control; *F*_(2, 14)_ = 0.94, *p* = 0.41; Fig. 4m–o).

### HtauE14 infused into the LC of 14–16-month-old rats results in impairment in simple odor discrimination learning and LC neuron loss 5–7 months post-infusion

To compare the potential differential effects of early onset vs. late onset tauopathology originating in the LC, we conducted an experiment in rats infused with AAV at an older age (14–16 months old; Fig. 5a). Six months post-infusion, htauE14 rats demonstrated more severe behavioral deficiency than rats infused at a young age (2–3 months old) had exhibited after a similar post-infusion interval. The older infused htauE14 rats were impaired in learning the first simple, dissimilar odor discrimination task (no. of blocks to reach learning criterion 7 ± 0.62, *n* = 7 in control vs. 11 ± 1.7, *n* = 4 in htauE14, *t* = 2.71, *p* = 0.024; Fig. 5b). There was also significant LC cell loss in the older infused htauE14 rats (917 ± 57.8/mm^2^, *n* = 6) compared to their age-matched controls (1181.7 ± 55.7/mm^2^, *n* = 5, *t* = 3.28, *p* = 0.01; Fig. 5c–e). As in the younger infused rats, NET fiber density in the piriform cortex of the older infused htauE14 rats (131.2 ± 13.7 μm/10,000 μm^2^, *n* = 6) was significantly lower than in control rats (208.5 ± 22.4 μm/10,000 μm^2^, *n* = 6, t = 2.94, *p* = 0.015; Fig. 5f–h). DBH fiber density was also significantly lower in the htauE14 rats (121.7 ± 7.4 μm/10,000 μm^2^, *n* = 6) compared to control rats (201.7 ± 13.0 μm/10,000 μm^2^, *n* = 6, *t* = 5.36, *p* = 3.21E−4; Fig. 5i–k). Additionally, there was an increased density of Iba-1^+^ microglia cells in the piriform cortex in htauE14 rats (htauE14 197.3 ± 6.2/mm^2^, *n* = 6 vs. control 151.7 ± 9.3/mm^2^, *n* = 6, *t* = 4.10, *p* = 0.002; Fig. 5l–n). There was no evidence of tangles as indexed by Thioflavin-S staining in the older infused htauE14 rats (Additional file 5).

Finally, in old rats, we observed neuron to microglia spread as well as transneuronal spread (Figs. 1e–g and 6). HT7^+^ cells were observed both in the genu of the corpus callosum and in the overlying sensory cortex (Fig. 6a–c). Co-labeling with the microglia marker Iba-1 was observed in both the corpus callosum (Fig. 6d1–d3) and sensory cortex (Fig. 6e1–e3). Astrocyte marker GFAP showed no co-localization with GFP cells (Fig. 6f1–f3). However, co-labeling with NeuN was also observed (Fig. 6 g1–g3). Additional file 6 shows zoom in images of individual cell types.

## Discussion

The rats expressing hyperphosphorylated human tau in the LC in these experiments show similarities to the human pretangle stages described by Braak [1, 3]. They exhibit abnormal tau somatodendritic mislocalization; transport of abnormal tau along LC fibers as far as the olfactory bulb by the first time point examined, 3–4 months post-infusion; and transfer of abnormal tau to the brain stem neurons, including the raphe neurons, by the same time point. Since abnormal tau is expressed in the LC neurons as early as 1 week after infusion, its influence on LC function would have been long-standing by 3–4 months post-infusion.

Despite LC pretangle tau expression, distribution, and transfer, rats 3–4 months post-infusion were not impaired on the difficult similar odor discrimination task that is sensitive to attenuation of LC input to piriform [13]. This parallels the human condition, since pretangle LC stages appear in people between 10 and 30 years of age, including transfer to the subcortical nuclei, most consistently, the raphe nuclei, with further extension along the LC axons to the terminal regions from 30 to 50 years of age. During this time frame, there is little evidence of olfactory dysfunction [14].

Congruent with our hypothesis that early olfactory deficits in rats, as in humans, are predictive of pretangle progression toward AD, we find that 7–8 months post-infusion, there are two changes in rats expressing abnormal LC tau. First, LC htauE14 rats are now impaired in difficult olfactory discrimination, though they acquire the simple odor discrimination normally. The impairment in difficult odor learning is not due to the inability to detect a difference in the odor mixtures that were used, as rats showed normal detection in the habituation/dishabituation task with the same odor pair. This is also consistent with a requirement for pattern separation-dependent associative memory to solve the difficult odor discrimination task. While pattern separation itself implies the ability to remember a distinct input among other similar inputs, in the brain, we have previously shown that only associative learning of pattern separation using similar odors is indexed by increased distinctiveness of neuronal ensembles representing the two odors in the rat piriform cortex [38].

Second, there is a significant reduction of LC fiber density in the piriform cortex as measured by NET or DBH fiber density. There is also upregulation of β1-adrenoceptors in the piriform cortex. This compensatory increase of β-adrenoceptor density with LC fiber loss is well known [43]. The increase in β-adrenoceptors may be compensatory but may also lead to deleterious effects. For example, CA3 hyperexcitability, a brain aging change associated with cognitive impairment in rodents and humans [44, 45], could be linked to increases in β-adrenoceptor activation [46].

We suggest the difficult olfactory discrimination impairment is driven by the reduction in LC fibers, since a pharmacological cocktail blocking LC input to rat piriform prevents acquisition of a difficult, but not simple, odor discrimination [13]. While it has been thought that olfactory identification deficits with aging are likely related to the sensory changes seen generally in peripheral tissue, e.g., olfactory epithelium, Devanand has suggested that these changes are early indicators of pretangle stage progression [16]. In a study examining olfactory, visual, and auditory aging changes related to cognitive decline, only the olfactory identification deficit predicted decline [21]. Our data also suggest that refinement of olfactory identification tasks, for example, by increasing odorant similarity during associative learning may lead to improved detection of preclinical Alzheimer’s disease as proposed by Hsieh et al. [47].

The reduction in LC fiber input here is consistent with the reports that norepinephrine (NE), the primary LC neurotransmitter, is reduced in AD [48]. In AD, measures of LC fiber density using DBH also demonstrate reduced NE fiber density [49]. Since tau facilitates axonal transport, it is not surprising that sustained production of abnormal tau eventually leads to reduced axonal support and results in axonal degeneration.

Similar to our findings here, a transgenic rat AD amyloid model in which hyperphosphorylated tau is also generated early in LC neurons exhibits a reduction in NE fiber density, in this case, in the allocortex and medial prefrontal cortex [50]. In the transgenic model, as in the present model, there was no change in LC neuron number with the reduction in LC fiber density. As here, this is congruent with the preclinical human data. LC neurons are not lost in AD until Braak’s stage III, when memory problems appear [37, 51]. In the transgenic rat model, rats showed deficits in spatial reversal learning. An exciting feature of the transgenic rat study was the ability of chemogenetic LC activation to eliminate the cognitive impairment thought to be associated with LC pretangles. Thus, LC activation, even in the presence of hyperphosphorylated tau, has the ability to contribute positively to learning and memory and may provide a key target for therapeutic intervention as proposed earlier [52]. It would be of interest to examine the effects of LC activation in the present paradigm.

While consistent with the present results, the transgenic model cannot rule out a contribution of amyloid, since it is the fundamental feature of these transgenic rats and is produced in concert with the observed LC hyperphosphorylated tau. However, the present study underscores the ability of abnormal LC tau alone to drive pathology and cognition in preclinical AD as proposed by Braak.

The availability of PET markers for quantifying NET fiber density in the human brain means LC fiber density could be assessed in imaging studies [53–56]. An aging-related decline in LC NET [54] consistent with LC volume loss with age in humans [4] has been observed. This methodology would permit a test of the prediction from the LC hyperphosphorylated tau rat models that pretangle LC tau eventually induces regression of LC axonal innervation in some, or all, cortical structures. The rat models suggest this change may be one of the earliest biomarkers of pretangle progression to AD.

In the last experiment, we showed the time course of deleterious pretangle effects is influenced by the age of the rat at initiation. The ability to manipulate the initiation of pretangle stages longitudinally is a strength of the model. Braak’s human data suggest there is a long time window associated with pretangle progression. When rats were infused with the htauE14 gene at 14–16 months, their olfactory learning impairment 6 months later is more dramatic than that seen in young adult infused rats. They are impaired in acquiring, even, a simple odor discrimination. The importance for odor in a rat’s life suggests difficulty in simple odor memory acquisition may index the beginning of impairments in daily living activities from a rodent point of view or may correspond to the beginning of mild cognitive impairment for these animals. This would parallel Braak stages III–IV when LC cell loss begins to be seen in humans. However, the limitation of this study is that our model does not mimic the tangle formation occurring in the human Braak stages.

Consistent with the relationship between mild cognitive impairment and LC cell loss in humans, LC cell loss was seen in the rats infused at 14–16 months when simple odor acquisition became difficult. There was also an increase in piriform microglial density. Increases in microglia density have been reported with AD [57, 58]. Thus, age significantly modulates the progression and expression of pathology associated with LC pretangles. In our model, older rats exhibit cognitive deficits, despite the absence of tangles. This supports the suggestion that soluble hyperphosphorylated tau may be the more toxic species and be driving deleterious AD changes [59]. The deleterious effects of pretangles only occurred in aged rats, however, congruent with the observation that AD is an aging disorder.

Another important observation in the older rats is the uptake of htauE14 by microglia in the corpus callosum and overlying cortex and the transfer of htauE14 to the cortical neurons. Uptake of hyperphosphorylated tau by microglia has been reported in AD [60]. The concentration of microglia containing htauE14 in the corpus callosum may relate to the white matter changes reported in AD [61–64]. Microglia could play a role in the spread of hyperphosphorylated soluble tau [65, 66]. In both young and older rat brains, hyperphosphorylated soluble tau spreads to neurons. This is likely to relate to the extracellular release of tau and may, or may not, involve exosomes [67]. The early transfer to the raphe neurons appears pathway specific in humans and in our rat model. Later transfer may become increasingly diffuse since ultimately most of the forebrain expresses neuronal hyperphosphorylated tau and tangles in late AD.

The present observations suggest that the LC supplies abnormal tau for both neuronal and microglial uptake. Similar transfer has been observed in another recent rodent hyperphosphorylated tau model [67]. While these observations do not rule out prion-like transfer, they suggest that such spread is likely to co-occur with spread originating from the initial source of abnormal tau, and in our model and in humans, that is the LC.

### Strengths of the htauE14 model

The htauE14 model provides control of selective expression of a functionally persistent phosphorylated human tau in LC neurons. The distribution of htauE14 corresponds to the distribution of pretangles described in Braak’s study [1] of human brains from 1 to 100 years of age including its early transneuronal spread to the raphe neurons.

Age strongly modulates the htauE14 pathophysiology with more serious effects of htauE14 expression in older rats. There is no learning deficit or loss of LC neurons in young adult rats, similar to the lack of either a behavioral or pathological signature in young adult humans. In the equivalent of rat late middle age, an impairment in difficult olfactory discrimination learning appears concomitant with LC axonal regression in the olfactory piriform cortex. Difficulty with simple olfactory discrimination learning only appears with htauE14 expression in older rats and with LC neuron loss, providing functional and pathological parallels to symptomatic AD.

The htauE14 model provides a testbed for therapeutic strategies to increase the health of LC neurons and/or to prevent or slow the pathological and functional sequelae of pretangle spread from LC. Importantly, the spread of htauE14 to other neurons and microglial cells means this model can be used to elucidate the mechanisms underlying the selective spread of pathology in preclinical AD.

Predictions based on the htauE14 model can be tested in humans using PET-NET imaging. A prediction of the present dataset is the reduction of LC axonal innervation in olfactory areas indexed by NET when deficits in olfactory discriminative memory appear. If validated, this may provide the earliest biomarker of LC pretangle-associated brain changes.

### Limitations of the htauE14 model

While the data suggest that deficiencies in associative olfactory memory are an early indicator of a declining LC functional support due to NE axonal retraction in the piriform cortex, deficits in olfactory identification memory are not specific for AD. In particular, similar deficits in olfactory identification are also seen in individuals who go on to develop Parkinson’s disease [68–70], although other forms of palsy are not associated with olfactory identification deficits [71]. Thus, testing olfactory identification deficits, while possibly useful in differentiating AD from other causes of later memory decline, does not provide a selective signature of preclinical AD.

Nonetheless, it appears that there is a link between the two neurodegenerative conditions that relates to failure in LC function [51, 72–75]. Abnormal α-synuclein accumulation in the LC is one of the earliest features of Parkinson’s disease, and the accumulation of this protein in LC neurons precedes its appearance in dopamine neurons [72]. Both preclinical AD and Parkinson’s disease may be associated with olfactory discrimination impairments for the same reason: the vulnerability of LC axonal support to abnormal protein accumulation.

Individuals with olfactory discrimination impairments are not aware of their dysfunction [76], yet it predicts later more problematic neurodegenerative-related decline. Olfactory discrimination impairments are also variable in their manifestation with repeated testing [77] suggesting a general associative olfactory memory decline rather than a specific sensory deficit. The common olfactory identification deficits associated with preclinical Alzheimer’s and Parkinson’s disease argue that an early intervention that would promote LC axonal health, see, for example, the work of Nakamura’s group [78], could be beneficial in the amelioration of both conditions.

A second limitation of the htauE14 model is related to the use of pseudophosphorylated tau as a stand-in for naturally occurring pretangle persistently phosphorylated tau in humans. Pretangle tau is normally indexed by immunohistochemistry using phosphorylation-specific antibodies post-mortem such as the AT8 antibody. These phosphorylation site-specific antibodies typically do not recognize sites that are pseudophosphorylated [42]. We saw no evidence of AT8 reactivity in our tissue, although we could demonstrate that pseudophosphorylated human tau was being expressed. The lack of post-mortem reactivity to antibodies for tau phosphorylation in the present model also argues that the six rat tau isoforms were not altered by exposure to the abnormal human tau construct to become abnormally phosphorylated. While there is evidence that pseudophosphorylated htauE14 is functionally similar to biologically phosphorylated tau [34], mechanistically, it may not behave in the same way [79]. Additionally, the interactions when there are multiple pseudophosphorylated sites appear complex [80].

The complete early pattern of persistent tau phosphorylation in pretangles in not known, but as reviewed, proline-directed sites are implicated. In the htauE14 model here, there is as yet no evidence that pseudophosphorylated tau becomes aggregated similar to other reports for multi-site pseudophosphorylated tau [81, 82]. Consistent with our observation, pseudophosphorylation at site S422 (one of the E14 sites) may protect against aggregation [83]. However, multiple pseudophosphorylation site effects on tau aggregation cannot be predicted from single-site properties [84]. Soluble tau is proposed to be more deleterious than tangle aggregates [85], however, and pretangle soluble tau is produced throughout the course of AD [86].

While htauE14 interferes with synaptic function in the same way as biologically phosphorylated mutant fronto-temporal dementia tau [34], mutant fronto-temporal dementia tau does not normally occur in the LC [87] and, thus, is a poor model of pretangle AD tau. Other attempts to model LC pretangle tau have failed. In mice, the fibrils of AD tau placed adjacent to the LC induced tau hyperphosphorylation in the LC neurons, but the spread of that hyperphosphorylated tau did not show the patterns seen in humans [88].

It will be important to characterize the nature of the present LC pretangles in terms of whether and/or when oligomers form, whether or not insoluble tangles appear, and whether beta-amyloid modulates htauE14 spread and functional outcomes. We also do not know if there is overexpression of total tau with the addition of htauE14. In other models, overexpression of LC wild-type protein alone induces pathology [89].

## Conclusions

Our animal model suggests, for the first time, that Braak’s hypothesis that human AD originates from the LC pretangle tau stages is plausible. Our results provide evidence that pretangle spread can occur through abnormal LC tau production, itself, rather than only through prion-like propagation. LC pretangle progression here generates both preclinical AD pathological changes and cognitive decline in the absence of amyloid. The odor discrimination deficits are similar to human odor deficits seen with aging and preclinical AD. When initiated in aged rats, pretangle stages progress rapidly and cause LC cell loss. These age-related outcomes are associated with more severe learning impairment consistent with memory decline in Braak stages III–IV when LC neurons are also lost.

## Additional files

Additional file 1: Locus coeruleus (LC) targeting reconstruction. Targeting success of 59 infusion sites from 30 rats are presented across 5 coronal planes along the rostro-caudal axis of the LC. For each hemisphere, infusion site was marked as the midpoint of maximum concentration of beads and represented in the corresponding plane in the cartoon. Black dashed outlines indicate LC. Coordinates are based on the atlas of Paxinos and Watson (4th edition). (PDF 431 kb)

Additional file 2: Locus coeruleus htauE14-GFP axonal projections 3 months post-infusion. GFP fibers were observed in the dentate gyrus (DG; a1-a2), hippocampal CA3 (b1-b2), olfactory bulb (OB; c1-c2), and piriform cortex (PC; d1-d3 showing co-labeling of DBH fiber (d1&d3, red) and GFP fiber (d2&d3, green), indicated by arrows). Scale bars, 50 μm. (PDF 980 kb)

Additional file 3: Tryptophan hydrolase (TPH) expression in the brain stem. a-b. Examples of TPH staining of raphe neurons at the level of the 4th ventricle. Arrows indicate areas of TPH^+^ cells. Scale bar, 50 μm. (PDF 632 kb)

Additional file 4: AT8 antibody failed in htauE14 pseudophosphorylated tau tissue despite human tau expression indexed by HT7. a. An example of HT7 staining in the locus coeruleus (LC) of a TH-CRE rat 3 weeks following htauE14-AAV infusion. Arrows indicate HT7^+^ cells. b. An adjacent LC slice showing no AT8 staining. Scale bar, 50 μm. (PDF 648 kb)

Additional file 5: No tangle formation is apparent in htauE14 brains. a1–a2. Ten min 90 °C heat treatment quenches GFP signal in a htauE14-EGFP brain. a1 shows GFP^+^ locus coeruleus cells without heat treatment, and a2 shows GFP quenching with heat in another section of the same brain. b–c. Thioflavin staining of neurofibrillary tangles in a TgCRND8^1^ mouse (gift from Dr. Bennett at University of Ottawa) section without heat treatment (b1–b2) and with heat treatment (c1–c2). Heat treatment does not prevent the detection of thioflavin-positive tangles. Arrows indicate stained tangles. b2 and c2 are enlargements from the red square areas in b1 and c1 correspondingly. d. Thioflavin staining in nonTg control mouse tissue showing no tangle formation. e–g. No tangle formation in a 21-month-old htauE14 rat 7 months post-infusion. e1–e2, Locus coeruleus; f, hippocampus; g, piriform cortex. h–j. No tangle formation in an 11-month-old htauE14 rat 8 months post-infusion. h1–h2, Locus coeruleus; i, hippocampus; j, piriform cortex. All tissues in panels e–j were treated with heat before thioflavin staining. Scale bars, 100 μm. ^1^Granger et al. A TgCRND8 mouse model of Alzheimer’s disease exhibits sexual dimorphisms in behavioral indices of cognitive reserve. J Alzheimers Dis. 2016, 53(1), 757–73. (PDF 1211 kb)

Additional file 6: Neurons and microglia, but not astrocytes, show uptake of htauE14. a1–a3. An example of GFAP labeling in the old rat brain as in Fig. 6. b1–b3. A GFP and Iba-1 double-labeled cell. c1–c3. An example of an Iba-1 stained microglia in a young htauE14 rat in the same region as in b. Note retracted morphology of Iba-1 cell in b compared to c. d1–d3. A GFP and NeuN double-labeled cell in the old rat brain. Scale bars, 25 μm. (PDF 2568 kb)

## Data Availability

The datasets used and/or analyzed during the current study are available from the corresponding author on reasonable request.

## Abbreviations

AAV: Adeno-associated virus
AD: Alzheimer’s disease
AR: Adrenoceptor
AT8: Anti-tau 8
DAB: Diaminobenzidine tetrahydrochloride
DBH: Dopamine beta-hydroxylase
DIO: Double inverted open reading frame
GFP: Green fluorescence protein
HT7: Human tau 7
Htau: Human pseudophosphorylated tau
Iba-1: Ionized calcium-binding adaptor molecule 1
NET: Norepinephrine transporter
NeuN: Neuronal nuclei
OD: Optical density
PBS: Phosphate-buffered saline
PFA: Paraformaldehyde
ROD: Relative optical density
SD: Sprague-Dawley
TH: Tyrosine hydroxylase
TPH: Tryptophan hydroxylase
