# Supplementary material for: An experimental model of Braak’s pretangle proposal for the origin of Alzheimer’s disease: the role of locus coeruleus in early symptom development
Source: Alzheimers Res Ther. 2019 Jul 3;11:59. doi: 10.1186/s13195-019-0511-2 (PMC6607586; doi:10.1186/s13195-019-0511-2)
Supplement: Supplementary file 1 — Locus coeruleus (LC) targeting reconstruction. Targeting success of 59 infusion sites from 30 rats are presented across 5 coronal planes along the rostro-caudal axis of the LC. For each hemisphere, infusion site was marked as the midpoint of maximum concentration of beads and represented in the corresponding plane in the cartoon. Black dashed outlines indicate LC. Coordinates are based on the atlas of Paxinos and Watson (4th edition). (PDF 431 kb) [file 13195_2019_511_MOESM1_ESM.pdf]

Additional File 1

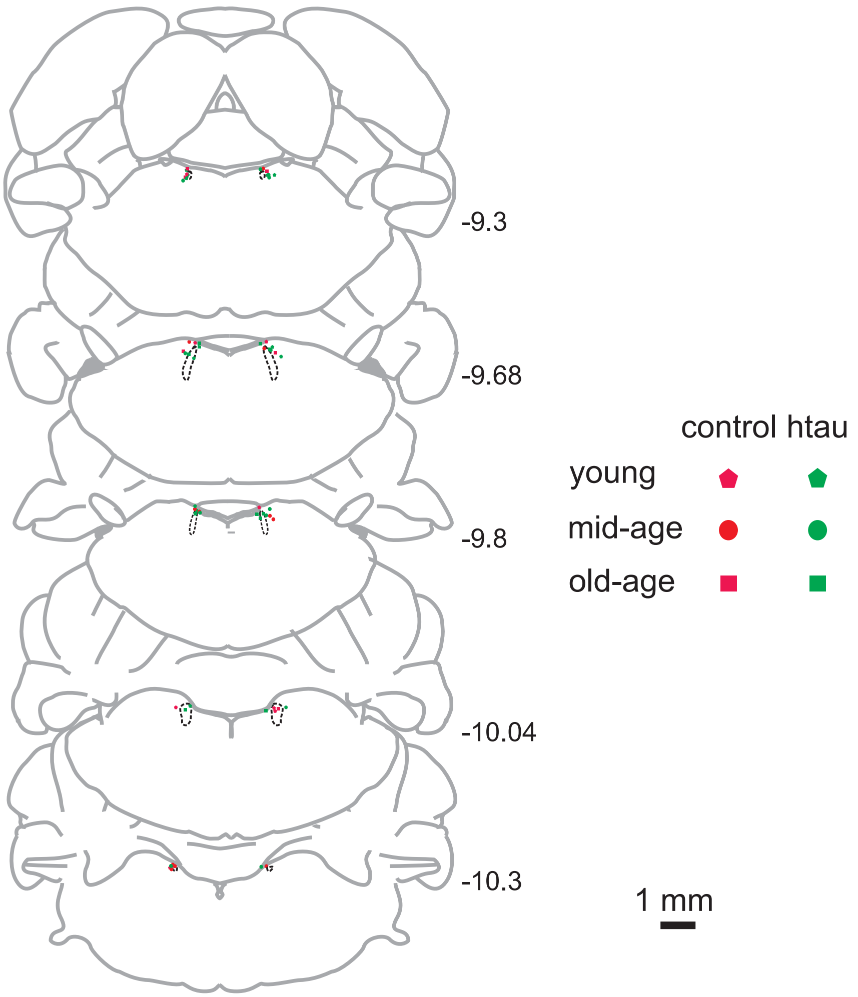

Locus coeruleus (LC) targeting reconstruction

Targeting success of 59 infusion sites from 30 rats are presented across 5 coronal planes along the rostro-caudal axis of the LC. For each hemisphere, infusion site was marked as the midpoint of maximum concentration of beads and represented in the corresponding plane in the cartoon. Black dashed outlines indicate LC. Coordinates are based on the atlas of Paxinos and Watson (4th edition).
