# Supplementary material for: An experimental model of Braak’s pretangle proposal for the origin of Alzheimer’s disease: the role of locus coeruleus in early symptom development
Source: Alzheimers Res Ther. 2019 Jul 3;11:59. doi: 10.1186/s13195-019-0511-2 (PMC6607586; doi:10.1186/s13195-019-0511-2)
Supplement: Supplementary file 2 — Locus coeruleus htauE14-GFP axonal projections 3 months post-infusion. GFP fibers were observed in the dentate gyrus (DG; a1-a2), hippocampal CA3 (b1-b2), olfactory bulb (OB; c1-c2), and piriform cortex (PC; d1-d3 showing co-labeling of DBH fiber (d1&d3, red) and GFP fiber (d2&d3, green), indicated by arrows). Scale bars, 50 μm. (PDF 980 kb) [file 13195_2019_511_MOESM2_ESM.pdf]

Additional File 2

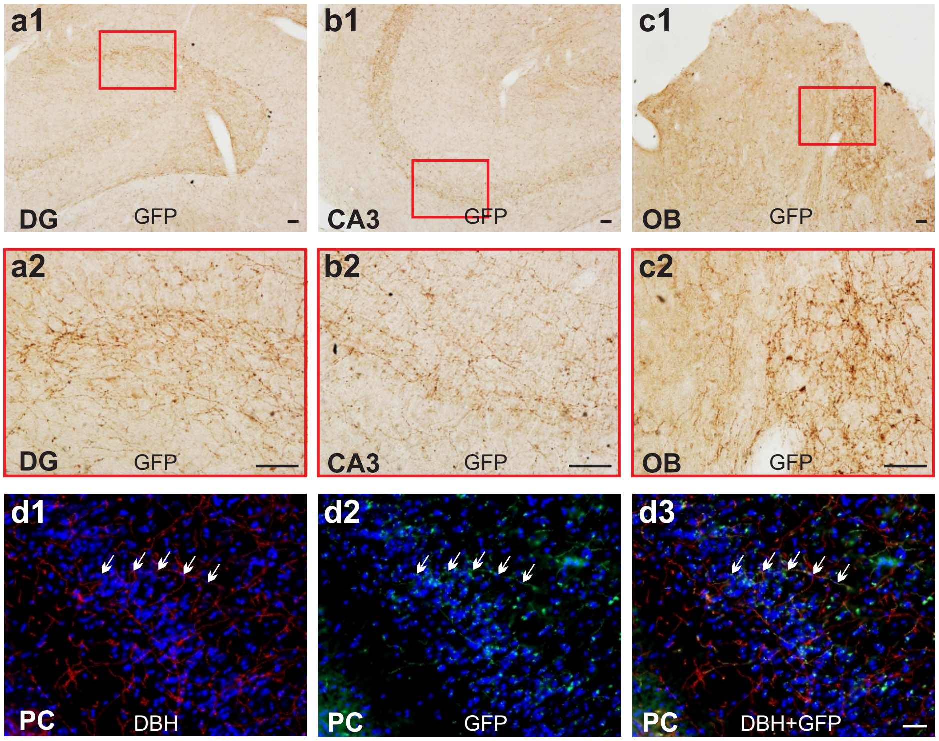

Locus coeruleus htauE14-GFP axonal projections 3 months post-infusion

GFP fibers were observed in the dentate gyrus (DG; **a1-a2**), hippocampal CA3 (**b1-b2**), olfactory bulb (OB; **c1-c2**), and piriform cortex (PC; **d1-d3** showing co-labeling of DBH fiber (d1&d3, red) and GFP fiber (d2&d3, green), indicated by arrows). Scale bars, 50 μm.
