# Supplementary material for: An experimental model of Braak’s pretangle proposal for the origin of Alzheimer’s disease: the role of locus coeruleus in early symptom development
Source: Alzheimers Res Ther. 2019 Jul 3;11:59. doi: 10.1186/s13195-019-0511-2 (PMC6607586; doi:10.1186/s13195-019-0511-2)
Supplement: Supplementary file 3 — Tryptophan hydrolase (TPH) expression in the brain stem. a-b. Examples of TPH staining of raphe neurons at the level of the 4th ventricle. Arrows indicate areas of TPH+ cells. Scale bar, 50 μm. (PDF 632 kb) [file 13195_2019_511_MOESM3_ESM.pdf]

# Additional File 3

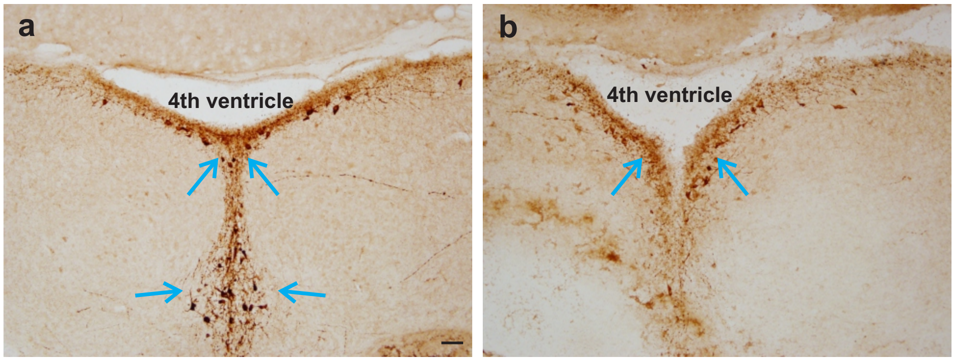


**Tryptophan hydrolase (TPH) expression in the brain stem**

**a-b**. Examples of TPH staining of raphe neurons at the level of the 4$^{th}$ ventricle. Arrows indicate areas of TPH$^{+}$ cells. Scale bar, 50 μm.
