# Supplementary material for: An experimental model of Braak’s pretangle proposal for the origin of Alzheimer’s disease: the role of locus coeruleus in early symptom development
Source: Alzheimers Res Ther. 2019 Jul 3;11:59. doi: 10.1186/s13195-019-0511-2 (PMC6607586; doi:10.1186/s13195-019-0511-2)
Supplement: Supplementary file 4 — AT8 antibody failed in htauE14 pseudophosphorylated tau tissue despite human tau expression indexed by HT7. a. An example of HT7 staining in the locus coeruleus (LC) of a TH-CRE rat 3 weeks following htauE14-AAV infusion. Arrows indicate HT7+ cells. b. An adjacent LC slice showing no AT8 staining. Scale bar, 50 μm. (PDF 648 kb) [file 13195_2019_511_MOESM4_ESM.pdf]

Additional File 4

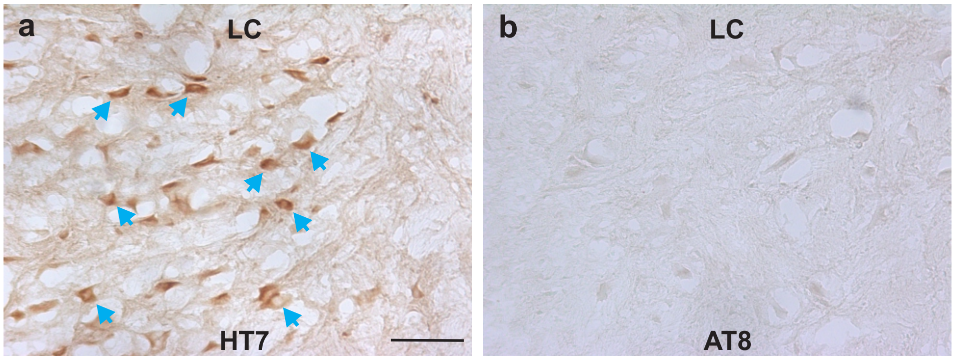

AT8 antibody failed in htauE14 pseudophosphorylated tau tissue despite human tau expression indexed by HT7

a. An example of HT7 staining in the locus coeruleus (LC) of a TH-CRE rat 3-weeks following htauE14-AAV infusion. Arrows indicate HT7^{+} cells. **b.** An adjacent LC slice showing no AT8 staining. Scale bar, 50 μm.
