# Supplementary material for: An experimental model of Braak’s pretangle proposal for the origin of Alzheimer’s disease: the role of locus coeruleus in early symptom development
Source: Alzheimers Res Ther. 2019 Jul 3;11:59. doi: 10.1186/s13195-019-0511-2 (PMC6607586; doi:10.1186/s13195-019-0511-2)
Supplement: Supplementary file 5 — No tangle formation is apparent in htauE14 brains. a1–a2. Ten min 90 °C heat treatment quenches GFP signal in a htauE14-EGFP brain. a1 shows GFP+ locus coeruleus cells without heat treatment, and a2 shows GFP quenching with heat in another section of the same brain. b–c. Thioflavin staining of neurofibrillary tangles in a TgCRND81 mouse (gift from Dr. Bennett at University of Ottawa) section without heat treatment (b1–b2) and with heat treatment (c1–c2). Heat treatment does not prevent the detection of thioflavin-positive tangles. Arrows indicate stained tangles. b2 and c2 are enlargements from the red square areas in b1 and c1 correspondingly. d. Thioflavin staining in nonTg control mouse tissue showing no tangle formation. e–g. No tangle formation in a 21-month-old htauE14 rat 7 months post-infusion. e1–e2, Locus coeruleus; f, hippocampus; g, piriform cortex. h–j. No tangle formation in an 11-month-old htauE14 rat 8 months post-infusion. h1–h2, Locus coeruleus; i, hippocampus; j, piriform cortex. All tissues in panels e–j were treated with heat before thioflavin staining. Scale bars, 100 μm. 1Granger et al. A TgCRND8 mouse model of Alzheimer’s disease exhibits sexual dimorphisms in behavioral indices of cognitive reserve. J Alzheimers Dis. 2016, 53(1), 757–73. (PDF 1211 kb) [file 13195_2019_511_MOESM5_ESM.pdf]

Additional File 5

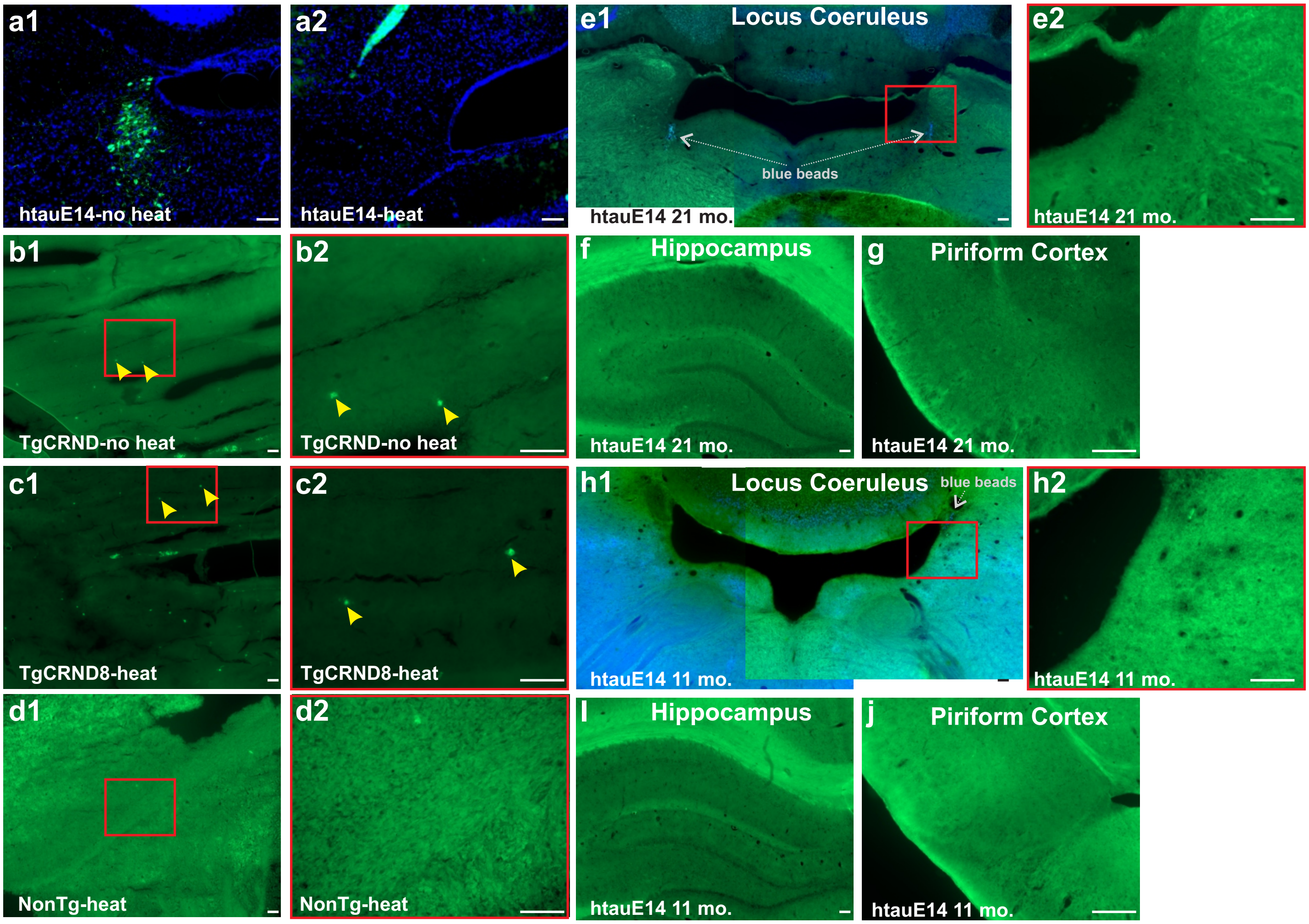

No tangle formation is apparent in htauE14 brains

a1-a2. Ten min 90°C heat treatment quenches GFP signal in a htauE14-EGFP brain. **a1** shows GFP$^+$ locus coeruleus cells without heat treatment and **a2** shows GFP quenching with heat in another section of the same brain. **b-c.** Thioflavin staining of neurofibrillary tangles in a TgCRND8[1] mouse (gift from Dr Bennett at University of Ottawa) section without heat treatment (**b1-b2**) and with heat treatment (**c1-c2**). Heat treatment does not prevent the detection of thioflavin positive tangles. Arrows indicate stained tangles. b2 and c2 are enlargements from the red square areas in b1 and c1 correspondingly. **d.** Thioflavin staining in a nonTg control mouse tissue showing no tangle formation. **e-g.** No tangle formation in a 21 month-old htauE14 rat 7 months post-infusion. **e1-e2**, Locus coeruleus; **f**, hippocampus; **g**, piriform cortex. **h-j.** No tangle formation in a 11 month-old htauE14 rat 8 months post-infusion. **h1-h2**, Locus coeruleus; **i**, hippocampus; **j**, piriform cortex. All tissue in panel **e-j** were treated with heat before thioflavin staining. Scale bars, 100 µm.

[1]Granger et al. A TgCRND8 mouse model of Alzheimer's disease exhibits sexual dimorphisms in behavioral indices of cognitive reserve. J Alzheimers Dis. 2016, 53(1), 757-73.
