# Supplementary material for: An experimental model of Braak’s pretangle proposal for the origin of Alzheimer’s disease: the role of locus coeruleus in early symptom development
Source: Alzheimers Res Ther. 2019 Jul 3;11:59. doi: 10.1186/s13195-019-0511-2 (PMC6607586; doi:10.1186/s13195-019-0511-2)
Supplement: Supplementary file 6 — Neurons and microglia, but not astrocytes, show uptake of htauE14. a1–a3. An example of GFAP labeling in the old rat brain as in Fig. 6. b1–b3. A GFP and Iba-1 double-labeled cell. c1–c3. An example of an Iba-1 stained microglia in a young htauE14 rat in the same region as in b. Note retracted morphology of Iba-1 cell in b compared to c. d1–d3. A GFP and NeuN double-labeled cell in the old rat brain. Scale bars, 25 μm. (PDF 2568 kb) [file 13195_2019_511_MOESM6_ESM.pdf]

Additional File 6

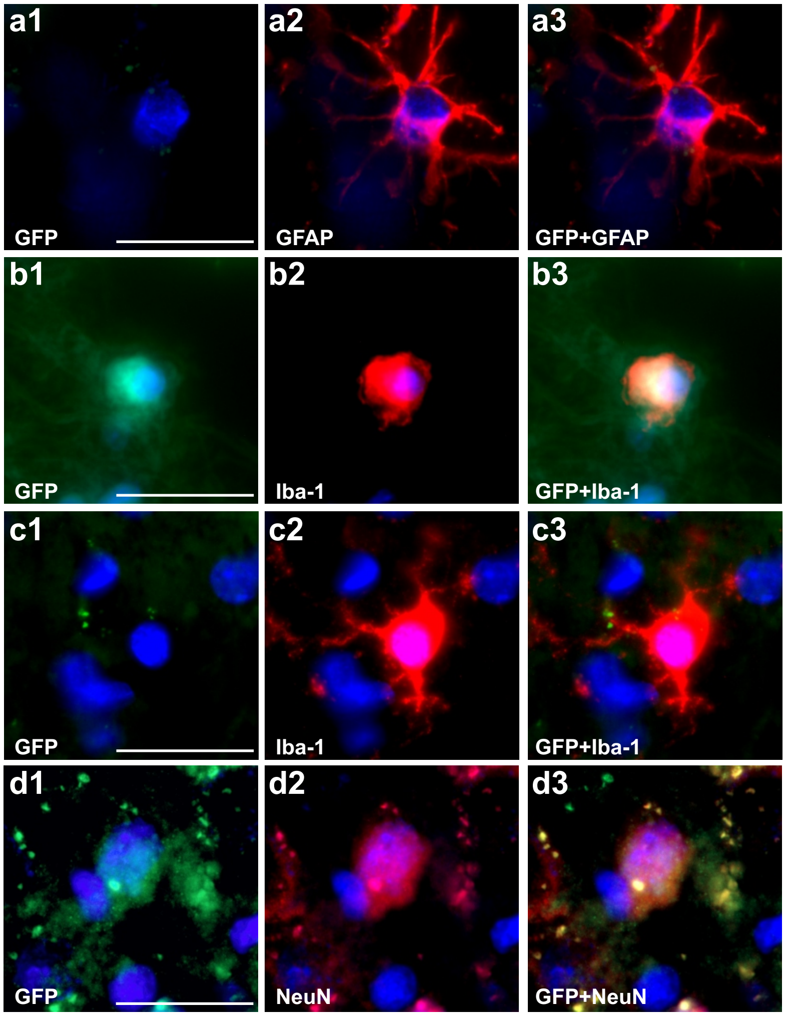

Neurons and microglia, but not astrocytes, show uptake of htauE14
a1-a3. An example of GFAP labeling in the old rat brain as in Figure 6. **b1-b3.** A GFP and Iba-1 double labeled cell. **c1-c3.** An example of an Iba-1 stained microglia in a young htauE14 rat in the same region as in **b**. Note retracted morphology of Iba-1 cell in **b** compared to **c**. **d1-d3.** A GFP and NeuN double labeled cell in the old rat brain. Scale bars, 25 μm.
